# Supplementary material for: Increased IL‐10‐producing regulatory T cells are characteristic of severe cases of COVID‐19
Source: Clin Transl Immunology. 2020 Nov 13;9(11):e1204. doi: 10.1002/cti2.1204 (PMC7662088; doi:10.1002/cti2.1204)

Supplementary table 1. Demographic, clinical and immunological data of healthy controls and COVID-19 patients. BMI, body mass index; CRP, C-reactive protein; HIV, Human Immunodeficiency Virus; M, male/F, female; n/a, not applicable or not available; WHO, World Health Organization. Laboratory parameters were obtained within 24 h of blood sampling for immune phenotyping.

			Aggregate statistics						Individual data													
			Healthy controls		Mild-moderate COVID-19		Severe COVID-19		Healthy controls						Mild-moderate COVID-19							
			Median	SEM	Median	SEM	Median	SEM	HC_001	HC_005	HC_006	HC_007	HC_008	HC_009	W_001	W_010	W_011	W_012	W_014	W_015	W_016	W_017
**Demographics**	Age		56	3	58	3	65	2	47	55	40	62	56	61	23	69	65	58	43	63	69	90
	BMI		n/a		23	1	24	1	n/a	n/a	n/a	n/a	n/a	n/a	32.42	26.83	25.39	22.72	22.66	33.06	27.45	24.39
	Sex		M=3, F=3		M=10, F=13		M=14, F=6		M	F	M	F	M	F	F	M	F	F	F	F	M	M
**Clinical condition**	WHO score		n/a		Score 3: 11, Score 4: 12		Score 5: 12, Score 6: 8		n/a	n/a	n/a	n/a	n/a	n/a	3	4	4	4	3	4	4	4
	Comorbitiies	Arterial hypertension	n/a		52% (12/23)		55% (11/20)		n/a	n/a	n/a	n/a	n/a	n/a	no	no	no	yes	yes	yes	yes	no
		Diabetes mellitus	n/a		9% (2/23)		20% (4/20)		n/a	n/a	n/a	n/a	n/a	n/a	no	no	no	no	no	yes	yes	no
		HIV	n/a		0% (0/23)		5% (1/20)		n/a	n/a	n/a	n/a	n/a	n/a	no	no	no	no	no	no	no	no
			Mean	SEM	Mean	SEM	Mean	SEM														
**Laboratory parameters**	CRP [mg/L]		n/a		88.35	12.79	165.67	22.07	n/a	n/a	n/a	n/a	n/a	n/a	21.40	67.70	58.30	18.80	1.30	100.50	193.10	197.80
	Whole blood count [x103/µL]		n/a		6.99	0.61	8.38	0.54	n/a	n/a	n/a	n/a	n/a	n/a	5.64	5.81	4.07	3.91	6.98	3.38	9.54	12.40
	Neutrophil count [x103/µL]		n/a		5.10	0.55	6.69	0.50	n/a	n/a	n/a	n/a	n/a	n/a	3.30	4.00	2.90	2.30	4.10	2.30	7.60	11.50
	Lymphocyte count [x103/µL]		n/a		1.28	0.16	1.05	0.13	n/a	n/a	n/a	n/a	n/a	n/a	1.70	1.10	1.00	1.00	2.40	0.70	1.10	0.40
**FlowSOM total cell populations [% of total]**	CD4+		44.15	4.48	48.82	3.11	46.18	3.50	56.05	45.85	33.51	44.45	34.71	63.85	62.01	42.42	70.54	39.93	54.70	63.09	87.72	15.23
	CD8+		16.24	1.13	12.12	1.36	10.79	1.27	16.35	26.95	21.02	23.24	25.05	15.64	9.40	12.55	14.70	14.97	20.27	17.32	5.17	32.15
	DNT		1.95	0.41	1.66	0.20	2.16	0.47	21.11	11.96	19.44	17.82	23.45	14.60	27.32	11.88	13.02	31.14	22.35	5.50	4.09	2.15
	CD19+ B cells		11.16	1.47	10.45	1.55	10.32	1.10	6.49	15.20	23.98	14.49	15.89	5.89	1.28	33.15	1.74	13.96	2.68	14.09	3.02	50.47
	CD14+ monocytes		12.54	2.32	13.61	2.42	15.15	3.00	0.00	0.04	2.05	0.00	0.89	0.02	0.00	0.00	0.00	0.00	0.00	0.00	0.00	0.00
**Manually gated total cell populations [% of total]**	CD4+		44.15	4.48	46.35	3.69	46.18	3.50	53.32	43.08	32.73	41.98	31.30	62.47	58.25	40.73	70.31	59.42	52.53	61.41	84.51	16.56
	Tconv		40.67	4.15	46.58	3.08	43.74	3.45	50.92	38.56	29.91	39.38	28.74	56.54	55.75	39.27	69.25	57.40	50.74	57.36	82.65	14.92
	Treg		2.98	0.45	1.92	0.20	2.09	0.16	2.07	3.88	2.38	2.36	2.15	5.02	2.05	1.23	0.80	1.52	1.52	3.51	1.56	1.50
	CD8+		16.24	1.13	12.12	1.36	10.79	1.27	16.75	11.50	17.86	17.07	20.16	14.08	25.58	12.89	10.43	25.23	20.09	4.15	3.42	1.78
	DNT		1.95	0.42	1.66	0.20	2.16	0.47	3.17	0.93	1.96	1.52	3.38	0.75	2.98	0.70	1.44	1.48	2.18	0.86	1.07	0.39
	CD19+ B cells		11.16	1.47	10.45	1.55	10.32	1.10	10.50	18.40	11.90	10.80	7.50	7.88	2.77	4.14	9.26	0.37	11.10	7.56	1.38	8.70
	CD14+ monocytes		12.54	2.32	13.61	2.42	15.15	3.00	5.83	15.43	21.49	12.04	15.19	5.27	2.24	28.87	2.41	3.89	2.95	17.20	1.80	49.20
**FlowSOM CD4$^+$ cell populations [% of CD4$^+$FOXP3$^-$]**	Naive		46.21	5.88	53.24	3.18	52.24	2.99	53.80	46.65	58.13	57.76	16.05	44.89	54.34	52.37	79.87	49.21	42.83	39.41	79.28	49.14
	TEM		21.19	4.69	15.72	2.23	11.99	1.82	13.15	20.13	19.47	11.16	45.84	17.38	12.37	13.29	3.29	9.74	28.29	31.12	4.08	14.01
	TCM		2.51	0.33	5.40	1.01	7.86	1.54	2.31	3.87	1.35	1.95	2.49	3.07	3.95	6.32	0.86	0.92	4.01	4.93	6.78	7.76
	Naive CTLA4$^+$		5.15	0.97	5.17	0.78	7.08	0.83	7.89	5.76	3.38	7.27	0.91	5.67	3.55	6.97	7.37	6.51	1.12	3.36	4.61	4.67
	TEM TNFα		6.73	1.12	5.95	0.60	5.69	0.77	5.42	7.78	1.65	8.38	6.67	10.47	10.26	5.99	2.30	4.87	7.24	11.12	2.37	6.32
	TEM Th1 CTLA-4$^-$		6.40	1.09	3.89	0.67	3.42	0.74	6.09	6.27	7.00	4.65	11.56	2.82	5.46	2.24	1.32	12.63	11.84	2.24	0.20	3.36
	TEM IL-2$^+$		3.45	0.72	3.13	0.79	3.47	0.54	3.53	2.98	0.75	2.16	5.53	5.73	2.96	4.61	1.71	2.70	0.39	2.96	0.92	2.76
	TCM CTLA-4$^+$		2.71	0.25	2.56	0.27	2.48	0.29	2.31	1.67	2.44	3.25	3.20	3.40	1.91	3.82	0.46	3.88	0.79	2.04	0.86	4.01
	TEM PD1^{hi}		1.83	0.38	1.32	0.25	1.81	0.40	1.04	0.82	2.42	0.96	3.25	2.49	0.72	0.86	0.13	0.59	0.66	0.46	0.07	3.88
	TEMRA Th1		1.50	0.27	1.08	0.24	1.10	0.21	1.95	2.36	0.44	0.82	1.71	1.71	1.84	1.12	0.39	5.13	0.59	1.05	0.07	0.86
	TEM Th1 CTLA-4$^+$		0.45	0.09	0.69	0.12	0.72	0.15	0.76	0.42	0.20	0.29	0.27	0.73	0.33	0.39	0.07	1.51	0.72	0.33	0.20	1.32
	TEM TNFα$^+$CTLA-4$^+$		0.60	0.13	0.55	0.12	0.69	0.16	0.55	0.44	0.15	0.84	0.45	1.16	1.78	0.39	0.20	1.18	0.33	0.59	0.07	1.32
	TCM IL-2		0.12	0.04	0.56	0.23	0.69	0.24	0.29	0.11	0.02	0.18	0.04	0.09	0.39	0.99	0.59	0.66	0.07	0.26	0.20	0.13
	TEMRA		0.70	0.29	0.37	0.11	0.39	0.11	0.36	0.15	1.76	0.15	1.65	0.13	0.07	0.39	0.13	0.46	0.86	0.00	0.00	0.13
	TEMRA Th17		0.46	0.09	0.38	0.07	0.40	0.11	0.56	0.58	0.85	0.16	0.36	0.25	0.07	0.26	1.32	0.00	0.26	0.13	0.33	0.33
**FlowSOM Treg cell populations [% of CD4$^+$FOXP3$^+$]**	TEM CTLA-4$^+$		35.02	3.65	26.95	2.51	32.69	2.20	25.69	25.50	36.25	32.81	51.94	37.94	11.51	35.28	12.73	24.78	26.04	16.79	13.81	54.72
	TEM		22.08	1.65	16.87	1.88	13.87	1.26	15.31	26.56	23.06	27.00	19.75	20.81	13.02	13.40	7.27	10.00	31.51	22.45	10.45	13.58
	Naive CTLA-4$^+$		15.46	1.77	17.47	3.31	13.76	1.76	22.06	13.25	17.19	16.00	7.69	16.56	19.81	16.23	32.73	20.43	6.42	18.49	16.42	2.26
	naive CTLA-4$^-$		10.60	1.35	11.59	1.21	8.10	1.17	14.50	13.63	13.44	7.06	6.88	8.06	23.58	9.06	13.64	4.35	21.70	14.15	10.82	0.75
	TCM		5.75	0.81	8.12	1.09	7.83	0.87	5.38	9.81	5.13	6.31	4.00	3.88	4.53	9.06	9.09	4.35	7.74	10.19	26.12	5.47
	TCM CTLA-4$^+$		1.86	0.19	4.33	0.56	7.34	0.79	2.69	2.06	1.25	1.81	1.44	1.88	4.72	8.87	2.73	4.78	0.19	4.53	7.84	6.79
	TNFα-producing CTLA-4$^+$		2.35	0.52	4.80	0.77	5.06	0.88	4.25	1.44	0.56	2.94	1.50	3.38	6.23	2.64	15.45	11.74	1.51	5.47	7.46	4.53
	IL10-producing		0.52	0.12	3.45	1.52	6.40	1.12	0.88	0.44	0.25	0.25	0.38	0.94	1.32	0.94	1.82	3.04	1.13	2.08	2.61	5.09
	TNFα-producing CTLA-4$^-$		2.52	0.24	2.59	0.58	2.22	0.37	2.88	2.63	1.31	2.88	2.38	3.06	4.53	1.32	0.00	0.43	0.94	3.02	3.36	1.13
	IFNγ-producing CTLA4$^+$		0.72	0.12	1.50	0.51	1.03	0.17	1.00	0.75	0.19	0.63	0.69	1.06	0.94	1.89	2.73	12.17	1.13	1.51	0.37	3.77
	IFNγ-producing CTLA4$^-$		1.47	0.28	1.09	0.34	0.59	0.12	2.06	2.56	0.50	1.56	1.00	1.13	7.92	0.38	0.91	3.48	0.94	1.13	0.00	0.00
	HLA-DR$^+$		1.23	0.26	0.69	0.17	0.39	0.11	1.75	1.13	0.88	0.50	2.38	0.75	0.19	0.57	0.00	0.00	0.57	0.00	0.75	1.70
	IL-2-producing CTLA-4$^+$		0.44	0.22	0.53	0.20	0.72	0.22	1.56	0.25	0.00	0.25	0.00	0.56	1.70	0.38	0.91	0.43	0.19	0.19	0.00	0.19
**Manually gated IL10+ [% of CD4$^+$FOXP3$^+$]**	IL-10-producing		2.13	0.40	5.90	1.30	9.96	0.88	3.47	2.24	2.11	0.95	0.87	3.12	6.16	8.10	5.50	9.26	3.41	5.35	4.07	12.50
**FlowSOM CD8$^+$ cell populations [% of CD8$^+$]**	naive		38.47	9.48	29.08	3.31	36.34	5.20	36.96	17.51	35.44	75.27	6.69	58.96	67.07	20.85	52.60	6.28	37.74	24.33	28.36	14.70
	TEM		21.09	2.69	27.33	2.81	24.07	3.79	13.25	29.45	25.67	11.42	24.73	22.02	13.60	36.46	12.15	14.45	21.95	35.37	46.46	13.23
	TEMRA Tc1		19.42	5.34	14.72	1.81	14.73	2.57	30.24	16.93	22.55	3.96	39.42	3.42	7.26	6.34	13.83	20.91	15.24	6.28	4.48	31.71
	TEM Tc1		13.19	3.88	13.84	2.02	11.34	2.07	11.69	30.80	4.53	5.11	20.22	6.80	6.77	5.37	9.27	51.71	18.84	18.90	2.05	16.52
	TEMRA		4.36	1.45	5.72	0.93	6.37	1.33	3.71	2.02	10.62	1.05	7.56	1.22	2.68	1.22	4.35	3.17	2.38	4.88	2.80	5.24
	TEM TNFα$^+$		1.16	0.42	3.70	1.14	1.79	0.29	0.85	0.96	0.25	1.56	0.11	3.20	0.85	11.77	3.37	0.00	1.10	4.09	9.70	0.00
	TEM IL-2$^+$		0.40	0.17	1.82	0.56	1.57	0.45	0.38	0.42	0.04	0.29	0.02	1.24	0.67	10.18	1.83	0.37	0.06	1.40	2.61	0.24
	TEM Tc1/17		0.08	0.02	1.28	0.57	0.76	0.33	0.15	0.13	0.04	0.05	0.07	0.05	0.06	0.43	0.07	0.18	0.37	0.98	0.00	12.87
	TEM Tc1 IL-2$^+$		0.57	0.17	0.68	0.14	1.05	0.40	0.95	1.16	0.05	0.13	0.45	0.69	0.55	0.98	0.56	2.56	0.67	1.83	1.31	0.98
	Naive CTLA-4$^+$		0.49	0.13	0.50	0.13	1.06	0.28	0.67	0.24	0.27	0.78	0.04	0.93	0.18	2.20	0.70	0.18	0.18	0.43	0.75	0.00
	TCM CTLA-4$^+$		0.55	0.18	0.73	0.18	0.66	0.11	0.89	0.22	0.36	0.33	0.09	1.38	0.24	4.02	1.26	0.18	0.30	1.22	0.93	1.10
	TEM Tc17		0.22	0.07	0.60	0.20	0.25	0.10	0.25	0.16	0.18	0.04	0.60	0.09	0.06	0.18	0.00	0.00	1.16	0.30	0.56	3.41

			W_018	W_019	W_020	W_022	W_024	W_025	W_026	W_027	W_029	W_033	W_036	W_042	W_043	W_046	W_048	Severe COVID-19<br>ICU_002	ICU_003	ICU_005	ICU_006	ICU_012
**Demographics**	Age		39	58	83	67	61	53	58	54	81	43	58	53	71	58	74	53	43	71	74	63
	BMI		31.24	28.69	32.96	40.3	16.41	25.1	19.88	33.14	24.55	22.06	24.38	31.7	19.29	25.08	24.91	27.16	46.69	29.41	23.99	25.61
	Sex		F	M	F	M	F	M	M	M	F	F	M	M	F	F	F	M	F	F	M	M
**Clinical condition**	WHO score		3	4	4	3	3	4	3	4	3	3	4	4	3	3	3	5	6	6	6	5
	Comborbitiies	Arterial hypertension	no	no	yes	yes	yes	no	no	yes	yes	no	no	no	no	yes	yes	no	yes	no	yes	no
		Diabetes mellitus	no	no	no	no	no	no	no	no	no	no	no	no	no	no	no	no	yes	no	no	no
		HIV	no	no	no	no	no	no	no	no	no	no	no	no	no	no	no	no	no	no	no	no
**Laboratory parameters**	CRP [mg/L]		29.70	161.20	65.30	86.80	170.40	90.10	77.00	66.00	60.40	n/a	149.80	101.30	135.30	n/a	3.10	389.10	179.90	57.00	219.70	282.40
	Whole blood count [x103/µL]		12.25	7.09	10.47	5.12	7.43	5.56	8.92	3.46	3.80	9.87	8.67	5.33	2.60	n/a	9.40	11.68	10.20	8.48	9.91	5.97
	Neutrophil count [x103/µL]		7.90	6.00	7.00	3.20	6.80	4.50	6.30	2.20	2.40	7.00	7.10	3.60	n/a	n/a	n/a	9.90	5.20	6.40	8.50	4.90
	Lymphocyte count [x103/µL]		3.20	0.60	2.20	1.00	0.40	0.80	1.70	0.90	1.10	2.00	1.00	1.20	n/a	n/a	n/a	0.90	2.70	0.70	1.00	0.70
**FlowSOM total cell populations [% of total]**	CD4+		62.89	57.58	35.57	43.69	45.28	47.52	55.70	28.86	67.52	39.60	61.07	34.43	57.32	41.14	33.89	58.67	65.36	82.23	78.19	31.02
	CD8+		10.47	23.15	20.54	25.03	48.29	29.19	21.88	35.97	15.23	18.93	18.26	27.38	27.25	28.52	24.36	22.89	16.45	9.70	6.87	47.71
	DNT		17.99	8.59	14.23	10.13	4.10	13.96	12.95	7.32	13.89	24.90	14.30	18.72	9.66	13.49	13.56	9.52	11.69	6.39	10.66	11.87
	CD19+ B cells		8.66	10.67	29.53	21.14	2.33	9.33	9.46	27.85	3.36	16.38	6.24	19.26	5.77	16.04	28.12	8.92	6.51	1.69	4.28	9.40
	CD14+ monocytes		0.00	0.00	0.13	0.00	0.00	0.00	0.00	0.00	0.00	0.20	0.13	0.20	0.00	0.81	0.07	0.00	0.00	0.00	0.00	0.00
**Manually gated total cell populations [% of total]**	CD4+		61.64	53.99	36.29	39.80	42.28	42.11	53.79	31.20	62.59	37.21	57.38	35.52	55.94	37.00	32.39	55.21	61.66	71.60	76.82	26.16
	Tconv		58.99	48.43	34.66	37.29	40.12	40.76	50.67	30.33	60.78	35.76	55.83	34.21	53.31	33.82	29.11	51.90	58.89	68.53	73.44	24.17
	Treg		2.27	5.11	1.48	2.28	2.02	1.14	2.75	0.78	1.60	1.20	1.35	0.93	2.11	2.46	2.90	3.04	2.47	2.26	2.77	1.83
	CD8+		16.71	8.48	12.93	7.30	3.33	11.55	11.15	8.35	10.75	22.43	15.16	16.85	8.67	12.00	9.64	8.28	11.43	4.60	9.32	8.60
	DNT		1.31	1.53	1.75	3.08	1.59	1.41	0.62	1.96	1.56	3.87	1.80	1.31	0.82	0.54	3.91	1.05	0.42	9.37	1.60	1.28
	CD19+ B cells		5.01	9.56	4.98	6.36	35.20	12.90	10.80	21.50	6.66	10.60	11.90	16.90	18.80	8.72	15.20	10.10	9.94	6.01	2.48	6.86
	CD14+ monocytes		6.76	8.52	27.63	22.50	3.62	14.38	8.85	19.11	8.55	13.79	4.74	15.27	2.64	19.70	28.50	13.12	7.59	1.31	4.73	15.01
**FlowSOM CD4$^+$ cell populations [% of CD4$^+$FOXP3$^-$]**	Naive		56.58	26.58	36.58	44.08	44.12	61.51	46.58	83.62	67.50	60.66	73.88	54.41	51.84	28.22	41.91	36.63	73.59	52.29	65.76	43.47
	TEM		16.71	5.26	29.34	15.33	24.63	12.89	13.95	2.18	9.93	15.00	6.38	16.51	3.09	45.39	28.68	24.42	9.18	2.88	7.41	16.65
	TCM		3.82	6.12	23.82	9.47	2.94	1.32	3.55	0.38	3.68	7.89	1.64	10.66	1.45	4.14	7.70	4.74	1.12	2.71	1.24	4.29
	Naive CTLA4$^+$		1.97	9.21	1.25	4.14	2.94	6.91	9.61	5.02	5.99	1.64	8.75	2.63	17.70	0.33	2.63	2.21	4.47	14.24	8.53	5.41
	TEM TNFα		6.97	10.99	3.82	10.13	9.56	8.16	5.99	1.61	3.29	4.14	2.96	6.05	4.21	2.76	5.66	15.79	5.12	5.88	5.12	5.29
	TEM Th1 CTLA-4$^-$		6.45	2.24	1.45	3.42	1.10	3.42	6.78	2.08	3.03	2.70	2.04	2.96	1.38	8.49	2.63	2.42	1.24	1.29	0.88	11.94
	TEM IL-2$^+$		1.12	18.75	0.92	5.72	5.51	2.37	3.16	1.42	1.64	0.66	0.33	0.92	7.24	0.66	2.63	6.32	1.65	9.53	4.35	3.59
	TCM CTLA-4$^+$		2.43	5.86	1.32	2.89	3.31	1.58	3.68	0.85	2.83	2.43	3.09	3.03	2.11	1.71	3.88	2.53	0.82	2.53	2.88	1.59
	TEM PD1^{hi}		1.58	3.22	0.99	1.91	0.37	0.59	1.84	0.19	0.13	2.76	0.33	1.58	1.58	4.14	1.84	0.32	0.53	0.41	0.29	1.76
	TEMRA Th1		1.05	3.09	0.26	0.79	1.84	0.33	1.84	0.19	0.59	0.20	0.13	0.13	2.11	0.46	0.72	1.05	0.88	2.35	1.06	2.41
	TEM Th1 CTLA-4$^+$		0.66	2.04	0.07	1.18	0.74	0.00	1.84	0.09	0.20	0.46	0.13	0.46	1.64	0.86	0.72	1.16	0.24	0.65	0.35	1.76
	TEM TNFα$^+$CTLA-4$^+$		0.46	1.71	0.13	0.53	1.47	0.26	0.39	0.00	0.00	0.26	0.00	0.07	1.12	0.20	0.26	1.79	0.59	0.35	1.00	0.71
	TCM IL-2		0.07	4.41	0.00	0.07	0.37	0.20	0.13	0.09	0.46	0.00	0.07	0.00	3.75	0.00	0.00	0.32	0.18	4.29	1.06	0.18
	TEMRA		0.13	0.07	0.00	0.07	0.00	0.13	0.39	1.52	0.72	0.46	0.20	0.13	0.00	2.24	0.39	0.11	0.18	0.00	0.00	0.82
	TEMRA Th17		0.00	0.46	0.07	0.26	1.10	0.33	0.26	0.76	0.00	0.72	0.07	0.46	0.79	0.39	0.33	0.21	0.24	0.59	0.06	0.12
**FlowSOM Treg cell populations [% of CD4$^+$FOXP3$^+$]**	TEM CTLA-4$^+$		25.85	19.62	29.25	27.74	50.00	41.88	32.83	0.00	26.34	27.17	30.00	29.43	19.06	27.36	37.74	34.55	16.92	6.35	30.60	53.15
	TEM		21.89	8.30	30.75	20.00	7.14	16.23	10.94	0.00	23.05	20.00	18.87	16.04	6.60	33.77	32.83	14.55	19.92	7.94	10.47	16.04
	Naive CTLA-4$^+$		11.32	6.60	3.40	15.28	7.14	9.95	23.96	82.14	16.05	14.34	20.00	12.64	31.51	8.11	6.60	9.09	29.32	31.75	14.58	4.50
	naive CTLA-4$^-$		20.57	1.32	6.98	11.51	7.14	9.42	13.02	14.29	13.99	14.72	9.81	17.74	7.74	13.58	6.79	0.00	18.42	12.70	5.13	3.78
	TCM		4.53	4.91	15.66	13.02	7.14	4.71	3.77	0.00	7.00	12.64	4.72	9.06	3.96	10.57	8.49	7.27	5.64	4.76	4.31	2.70
	TCM CTLA-4$^+$		4.72	9.81	5.09	5.66	0.00	2.62	3.77	0.00	4.12	3.40	7.36	6.42	4.15	1.13	0.94	5.45	1.88	11.11	6.57	2.70
	TNFα-producing CTLA-4$^+$		3.21	8.87	0.75	1.70	7.14	6.81	2.64	3.57	4.53	1.13	2.08	2.45	8.49	0.57	1.51	12.73	3.01	9.52	14.17	2.70
	IL10-producing		0.75	36.42	1.51	1.51	0.00	1.57	1.89	0.00	1.65	0.94	3.77	1.32	9.43	0.19	0.38	7.27	1.13	9.52	3.90	7.57
	TNFα-producing CTLA-4$^-$		3.77	1.13	3.40	1.89	14.29	1.57	1.51	0.00	2.06	3.77	1.51	2.64	2.26	2.64	2.45	5.45	2.63	1.59	3.70	1.98
	IFNγ-producing CTLA4$^+$		1.70	1.32	0.00	0.57	0.00	0.52	2.45	0.00	0.41	0.57	0.75	0.57	1.13	0.00	0.00	1.82	0.38	3.17	0.82	1.80
	IFNγ-producing CTLA4$^-$		1.13	0.38	0.57	0.19	0.00	2.09	0.94	0.00	0.41	1.32	0.38	0.75	1.13	0.94	0.19	0.00	0.00	1.59	0.41	1.26
	HLA-DR$^+$		0.57	0.00	2.26	0.75	0.00	2.62	1.51	0.00	0.41	0.00	0.19	0.57	0.00	1.13	2.08	0.00	0.75	0.00	1.03	1.26
	IL-2-producing CTLA-4$^+$		0.00	1.32	0.38	0.19	0.00	0.00	0.75	0.00	0.00	0.00	0.57	0.38	4.53	0.00	0.00	1.82	0.00	0.00	4.31	0.54
**Manually gated IL10+ [% of CD4$^+$FOXP3$^+$]**	IL-10-producing		5.04	30.10	2.60	2.82	7.14	2.65	4.28	3.45	0.88	0.93	5.09	0.89	13.90	0.69	0.97	14.50	6.32	13.60	10.70	14.50
**FlowSOM CD8$^+$ cell populations [% of CD8$^+$]**	naive		47.32	38.14	10.55	15.91	18.18	13.41	26.28	45.26	13.97	55.30	40.24	17.01	27.62	17.20	30.43	71.90	88.53	33.05	73.59	4.85
	TEM		17.13	15.34	59.70	24.21	22.73	29.63	18.66	18.35	17.53	27.50	38.41	35.00	15.12	54.76	40.91	7.84	6.77	10.17	8.83	25.30
	TEMRA Tc1		17.26	9.87	6.28	22.26	9.09	17.01	25.18	20.49	30.78	4.21	7.13	20.55	27.68	4.02	10.67	3.27	0.72	15.25	4.16	18.61
	TEM Tc1		10.30	14.70	13.96	21.04	13.64	18.35	17.32	0.92	14.77	6.83	8.78	10.12	16.04	13.66	8.48	8.50	1.51	13.56	5.28	44.85
	TEMRA		5.24	4.55	6.46	3.23	4.55	5.06	6.40	9.48	21.29	4.45	2.99	16.46	5.55	3.84	5.30	1.31	0.88	5.93	1.62	1.29
	TEM TNFα$^+$		0.79	4.47	1.28	3.78	22.73	13.17	0.49	1.83	0.79	0.55	0.85	0.30	0.85	0.37	1.95	1.96	0.16	2.54	1.12	0.89
	TEM IL-2$^+$		0.12	5.26	0.61	1.71	9.09	2.26	0.37	2.45	0.40	0.00	0.06	0.00	1.28	0.06	0.73	3.27	0.40	6.78	1.55	0.54
	TEM Tc1/17		0.55	2.49	0.00	4.88	0.00	0.30	2.13	0.00	0.00	0.12	0.30	0.12	2.13	1.28	0.18	0.00	0.40	0.00	0.37	0.84
	TEM Tc1 IL-2$^+$		0.24	1.49	0.24	0.79	0.00	0.12	1.46	0.00	0.26	0.00	0.06	0.06	1.22	0.12	0.06	1.96	0.16	8.47	1.37	1.98
	Naive CTLA-4$^+$		0.12	1.14	0.06	0.43	0.00	0.12	0.61	0.61	0.00	0.37	0.98	0.00	2.20	0.12	0.18	0.00	0.00	3.39	1.62	0.30
	TCM CTLA-4$^+$		0.24	2.41	0.43	0.79	0.00	0.43	0.12	0.61	0.20	0.12	0.12	0.30	0.24	0.85	0.67	0.00	0.32	0.85	0.19	0.45
	TEM Tc17		0.67	0.14	0.43	0.98	0.00	0.12	0.98	0.00	0.00	0.55	0.06	0.06	0.06	3.72	0.43	0.00	0.16	0.00	0.31	0.10

			ICU_013	ICU_014	ICU_015	ICU_023	ICU_025	ICU_027	ICU_029	ICU_031	ICU_036	ICU_039	ICU_043	ICU_045	W_002	W_004	W_049
**Demographics**	Age		67	69	68	60	47	66	67	51	75	62	55	54	68	43	78
	BMI		38.73	24.84	29.07	30.86	39.18	23.78	30.67	33.08	23.67	29.77	22.23	29.05	27.76	35.92	24.22
	Sex		M	F	M	M	F	M	M	M	F	M	F	M	M	M	M
**Clinical condition**	WHO score		6	6	6	5	6	5	5	5	5	5	5	5	6	5	5
	Comorbitiies	Arterial hypertension	yes	yes	yes	no	no	no	yes	yes	no	yes	no	yes	no	yes	yes
		Diabetes mellitus	no	no	no	no	no	no	yes	yes	no	no	yes	no	no	no	no
		HIV	no	no	no	no	yes	no	no	no	no	no	no	no	no	no	no
**Laboratory parameters**	CRP [mg/L]		72.20	373.90	76.70	245.70	74.10	115.50	130.60	259.10	71.90	141.80	125.00	140.50	165.00	33.80	159.50
	Whole blood count [x103/µL]		7.41	14.92	7.28	10.69	10.11	6.30	8.65	9.05	9.06	6.71	6.76	7.02	8.36	4.86	4.16
	Neutrophil count [x103/µL]		6.30	13.50	4.40	9.00	7.10	5.40	6.40	8.30	5.60	5.00	5.80	5.40	7.90	3.30	5.50
	Lymphocyte count [x103/µL]		0.70	0.90	0.60	1.00	1.80	0.50	1.30	0.60	2.30	1.40	0.70	0.90	0.60	1.00	0.60
**FlowSOM total cell populations [% of total]**	CD4+		46.63	58.19	56.45	29.10	32.53	37.11	34.58	63.92	52.95	36.20	46.99	33.19	26.20	54.28	73.19
	CD8+		25.00	19.34	30.60	23.98	31.81	25.30	14.88	18.55	19.34	35.36	25.72	20.96	19.34	25.18	14.04
	DNT		24.52	10.36	5.78	17.35	25.12	7.11	8.55	14.94	13.98	11.93	24.76	9.16	4.88	10.96	11.81
	CD19+ B cells		3.86	12.11	7.17	29.52	10.54	30.48	41.87	2.59	13.67	16.45	2.53	36.69	49.58	9.58	0.96
	CD14+ monocytes		0.00	0.00	0.00	0.06	0.00	0.00	0.12	0.00	0.06	0.06	0.00	0.00	0.00	0.00	0.00
**Manually gated total cell populations [% of total]**	CD4+		44.95	55.26	42.86	26.76	29.76	33.36	30.77	56.10	51.44	33.91	44.51	37.80	24.45	49.14	71.06
	Tconv		42.70	51.72	41.40	24.72	27.14	30.66	27.35	53.69	48.45	32.42	41.66	34.93	23.76	47.08	70.28
	Treg		2.00	3.19	1.34	1.86	2.37	2.36	3.03	2.04	2.43	1.18	2.33	2.43	0.58	1.76	0.55
	CD8+		23.04	9.58	3.07	15.24	22.40	7.53	7.27	9.53	13.10	11.40	21.90	11.13	4.03	6.57	7.76
	DNT		1.81	0.61	4.24	1.25	1.43	0.50	1.18	4.22	1.65	1.04	2.32	1.06	0.37	4.85	2.90
	CD19+ B cells		18.20	13.20	16.30	10.10	16.10	21.40	6.98	10.40	7.69	8.27	15.40	5.09	4.53	11.50	5.80
	CD14+ monocytes		3.07	11.37	11.31	29.57	12.29	30.73	41.89	1.99	12.92	15.47	2.74	22.79	51.08	13.13	0.89
**FlowSOM CD4^{+} cell populations [% of CD4^{+}FOXP3^{-}]**	Naive		49.59	69.53	72.06	53.82	35.24	61.06	35.18	60.94	57.65	31.06	41.29	48.24	49.29	37.47	70.59
	TEM		5.35	3.29	3.88	10.41	20.88	4.41	16.12	5.06	8.24	32.94	13.00	21.06	7.65	20.06	6.82
	TCM		3.65	3.35	4.41	8.24	2.71	8.41	19.24	3.59	9.65	24.47	9.06	18.65	20.35	4.00	3.29
	Naive CTLA4^{+}		12.12	11.06	10.88	8.65	3.65	10.18	5.18	10.88	6.71	0.94	5.41	1.29	8.88	3.82	7.00
	TEM TNFα		5.94	2.18	2.06	2.65	7.65	3.76	6.06	4.53	3.00	5.00	8.82	2.59	4.76	13.53	4.00
	TEM Th1 CTLA-4^{-}		4.41	1.47	0.12	5.29	8.47	0.82	0.88	3.24	3.41	1.82	10.65	1.53	0.53	5.71	2.18
	TEM IL-2^{+}		5.59	3.24	1.47	1.65	5.35	3.18	1.82	6.41	1.59	0.47	2.94	0.24	1.24	6.76	2.06
	TCM CTLA-4^{+}		2.12	1.71	1.59	3.06	1.82	3.41	5.35	1.35	2.94	1.06	3.94	2.06	5.65	1.53	1.65
	TEM PD1^{hi}		0.94	2.06	0.88	3.29	3.82	1.59	7.59	0.41	3.53	1.35	1.41	3.76	1.35	0.82	0.12
	TEMRA Th1		3.00	0.47	0.29	0.71	3.00	0.82	0.18	1.29	1.18	0.06	1.47	0.06	0.00	1.59	0.12
	TEM Th1 CTLA-4^{+}		0.65	0.47	0.29	0.65	2.71	1.00	1.47	0.41	1.35	0.24	0.35	0.24	0.06	0.35	0.06
	TEM TNFα^{+}CTLA-4^{+}		1.24	0.29	0.29	0.24	2.71	0.12	0.76	0.47	0.12	0.06	0.47	0.12	0.06	2.12	0.24
	TCM IL-2		3.24	0.47	0.59	0.24	0.65	0.29	0.00	0.59	0.24	0.00	0.24	0.00	0.12	0.53	0.47
	TEMRA		0.00	0.18	0.00	1.00	1.18	0.00	0.06	0.35	0.12	0.18	0.88	0.12	0.00	1.47	1.18
	TEMRA Th17		2.18	0.24	1.18	0.12	0.18	0.94	0.12	0.47	0.29	0.35	0.06	0.06	0.06	0.24	0.24
**FlowSOM Treg cell populations [% of CD4^{+}FOXP3^{+}]**	TEM CTLA-4^{+}		27.62	31.38	28.77	44.00	29.17	39.38	39.69	29.85	27.38	41.85	42.77	39.23	31.69	31.77	27.71
	TEM		7.18	13.69	4.57	8.31	16.67	15.85	18.15	4.92	17.08	25.69	11.38	22.62	14.92	18.23	9.14
	Naive CTLA-4^{+}		14.64	16.62	14.61	10.46	21.25	7.08	4.31	21.54	10.62	2.77	9.69	5.38	11.38	13.54	22.00
	naive CTLA-4^{-}		6.63	6.46	10.50	3.08	12.92	5.69	3.23	5.54	19.08	2.46	4.62	15.85	6.31	9.12	10.57
	TCM		4.42	9.38	8.22	10.77	2.08	9.85	9.23	2.92	10.31	16.31	8.46	8.62	16.46	5.52	9.43
	TCM CTLA-4^{+}		8.29	11.69	10.05	10.46	2.92	11.69	13.54	7.85	7.85	4.77	10.62	3.54	9.08	3.31	3.43
	TNFα-producing CTLA-4^{+}		7.46	2.62	8.22	3.08	5.00	1.69	2.31	6.46	1.69	1.23	1.38	0.62	1.85	9.39	6.00
	IL10-producing		20.99	4.62	12.79	7.23	2.50	6.46	7.38	15.38	3.54	1.23	6.15	2.46	4.92	2.76	0.29
	TNFα-producing CTLA-4^{-}		0.28	1.38	2.28	0.92	2.08	0.46	1.23	1.69	0.46	3.08	1.38	0.62	2.15	4.14	6.86
	IFNγ-producing CTLA4^{+}		0.55	0.62	0.00	0.46	2.08	0.77	0.46	1.38	1.38	0.31	1.23	0.77	0.62	0.55	1.43
	IFNγ-producing CTLA4^{-}		1.10	0.46	0.00	0.62	1.25	0.46	0.00	0.92	0.15	0.15	0.92	0.15	0.15	0.55	1.71
	HLA-DR^{+}		0.55	0.00	0.00	0.15	1.67	0.00	0.15	0.00	0.00	0.15	0.46	0.15	0.00	1.10	0.29
	IL-2-producing CTLA-4^{+}		0.28	1.08	0.00	0.46	0.42	0.62	0.31	1.54	0.46	0.00	0.92	0.00	0.46	0.00	1.14
**Manually gated IL10+ [% of CD4^{+}FOXP3^{+}]**	IL-10-producing		6.32	13.60	10.70	14.50	6.32	13.60	10.70	14.50	6.32	13.60	10.70	2.03	6.05	5.57	5.09
**FlowSOM CD8^{+} cell populations [% of CD8^{+}]**	naive		42.52	36.14	68.30	14.50	30.59	28.76	25.35	61.04	13.96	14.90	11.44	24.46	25.64	36.55	20.69
	TEM		14.80	25.99	11.25	27.52	11.88	26.63	49.65	8.17	29.11	72.77	20.00	48.32	41.04	22.31	13.07
	TEMRA Tc1		11.14	15.94	2.25	29.36	36.04	15.45	3.81	13.32	26.39	1.09	32.48	5.30	9.50	13.94	36.53
	TEM Tc1		13.51	13.12	2.86	8.07	7.33	5.50	9.21	8.12	15.54	5.94	23.32	7.18	13.96	15.64	3.86
	TEMRA		0.89	2.03	6.34	18.32	12.33	6.19	3.86	1.93	11.34	1.93	8.32	12.43	4.26	3.85	22.38
	TEM TNFα^{+}		5.59	1.39	1.43	0.94	0.20	3.02	1.09	1.04	0.54	2.13	2.08	0.99	3.17	3.78	1.83
	TEM IL-2^{+}		7.08	1.88	1.02	0.35	0.20	2.33	0.20	2.38	0.30	0.05	0.69	0.10	0.20	1.63	0.45
	TEM Tc1/17		0.15	0.40	0.20	0.05	0.30	6.29	3.42	0.35	1.39	0.00	0.20	0.15	0.40	0.30	0.05
	TEM Tc1 IL-2^{+}		1.14	0.59	0.61	0.10	0.79	0.20	0.40	0.74	0.69	0.15	0.54	0.00	0.15	0.89	0.10
	Naive CTLA-4^{+}		2.13	0.94	4.91	0.35	0.20	1.98	0.64	2.03	0.20	0.35	0.20	0.54	0.74	0.37	0.30
	TCM CTLA-4^{+}		1.04	1.44	0.61	0.40	0.05	1.58	1.73	0.64	0.15	0.59	0.54	0.40	0.79	0.74	0.69
	TEM Tc17		0.00	0.15	0.20	0.05	0.10	2.08	0.64	0.25	0.40	0.10	0.20	0.15	0.15	0.00	0.05

Supplementary figure 1. Classification of lymphocytes. PBMCs were isolated from healthy controls (n=6), mild-moderate COVID-19 patients (n=23) and severe COVID-19 patients (n=20), stimulated and assessed through flow cytometry. **(a)** FlowSOM clusters (identified in Figure 1) were obtained based on CD3, CD4, CD8, FOXP3, CD19, CD14. Expression of each marker on each FlowSOM cluster is shown in histogram format and **(b)** imposed on a tSNE representation.

Supplementary figure 1

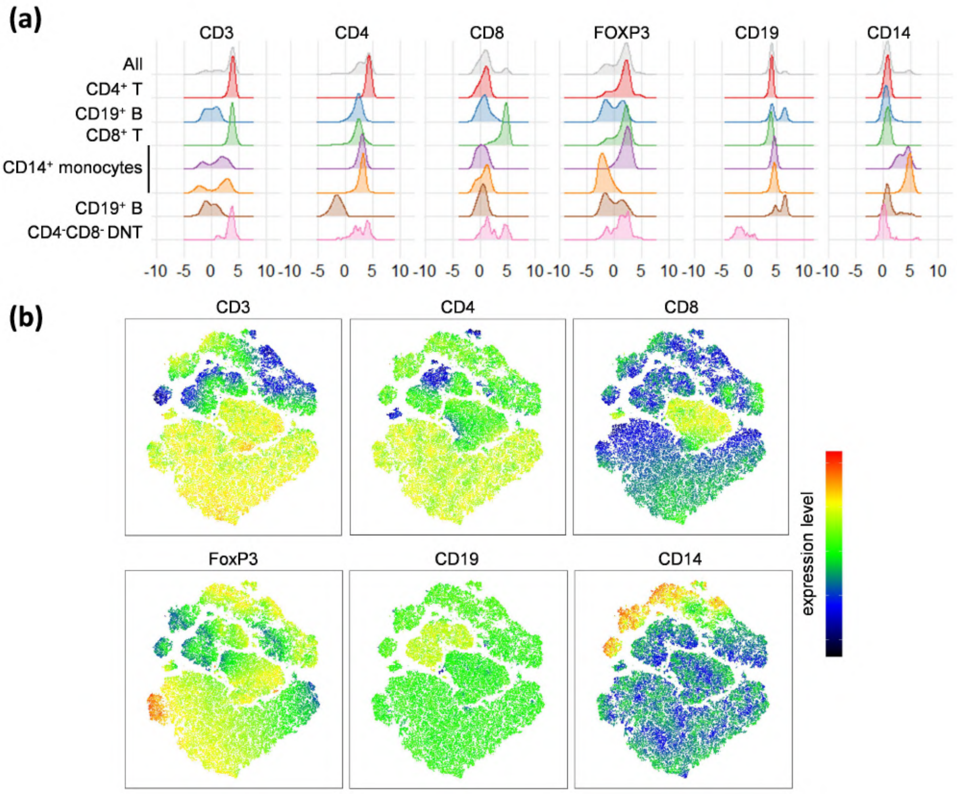

Supplementary figure 2. Classification of conventional CD4 T cells. PBMCs were isolated from healthy controls (n=6), mild-moderate COVID-19 patients (n=23) and severe COVID-19 patients (n=20), stimulated and assessed through flow cytometry. $CD3^{+}CD14^{-}CD4^{+}CD8^{-}FOXP3^{-}$ conventional T cells were manually gated in FlowJo. **(a)** FlowSOM clusters (identified in Figure 2) were obtained based on CD45RA, CCR7, 4-1BB, CD25, CTLA-4, HLA-DR, IFNγ, IL-2, IL-4, IL-6, IL-10, IL-17a, PD-1, RORγt, T-BET, TNFα. Expression of each marker on each FlowSOM cluster is shown in histogram format and **(b)** imposed on a tSNE representation.

Supplementary figure 2

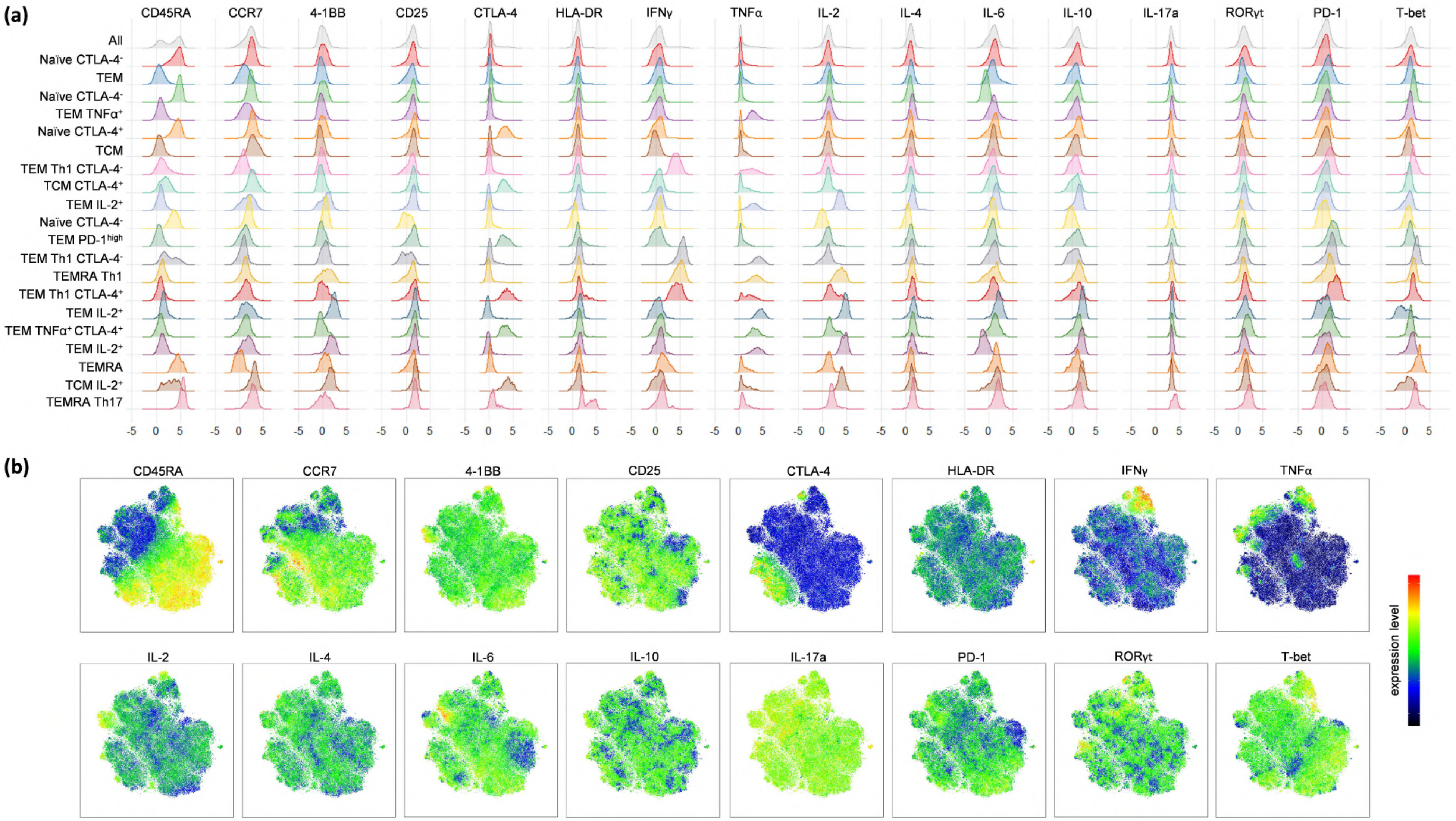

Supplementary figure 3. Classification of regulatory T cells. PBMCs were isolated from healthy controls (n=6), mild-moderate COVID-19 patients (n=23) and severe COVID-19 patients (n=20), stimulated and assessed through flow cytometry. $CD3^+CD14^-CD4^+CD8^-FOXP3^+$ regulatory T cells were manually gated in FlowJo. **(a)** FlowSOM clusters (identified in Figure 3) were obtained based on CD45RA, CCR7, 4-1BB, CD25, CTLA-4, HLA-DR, IFNγ, IL-2, IL-4, IL-6, IL-10, IL-17a, PD-1, RORγt, T-BET, TNFα. Expression of each marker on each FlowSOM cluster is shown in histogram format and **(b)** imposed on a tSNE representation. **(c)** Manual gating strategy for IL-10^+ regulatory T cells after gating on living single cells. **(d)** Correlation between expression of CD25 and FOXP3, as assessed by the geometric mean fluorescence intensity (gMFI), in total and IL-10^+ regulatory T cells of severe COVID-19 patients. The Spearman's rank correlation coefficient r as well as the significance of the correlation were calculated.

Supplementary figure 3

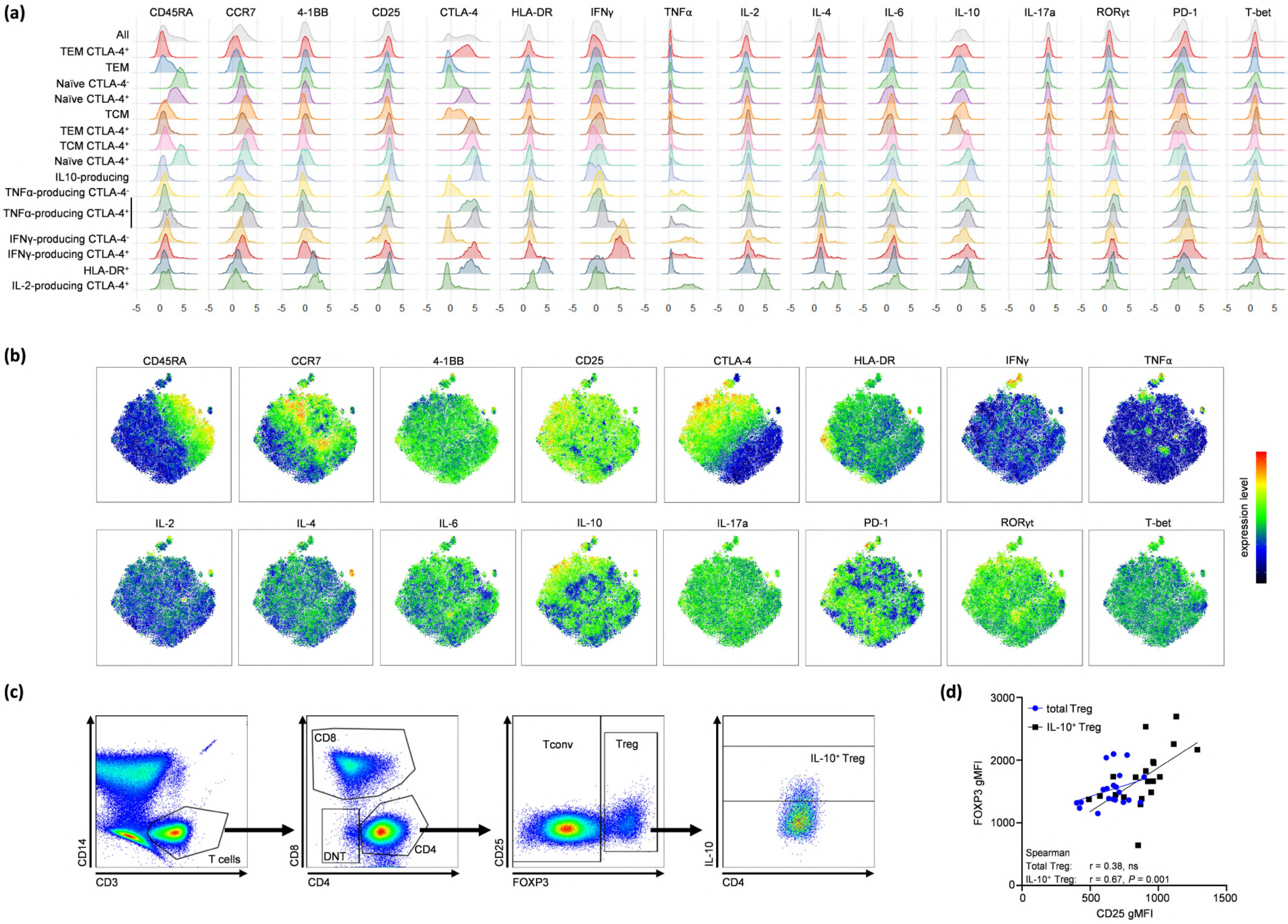

Supplementary figure 4. Classification of CD8 T cells. PBMCs were isolated from healthy controls (n=6), mild-moderate COVID-19 patients (n=23) and severe COVID-19 patients (n=20), stimulated and assessed through flow cytometry. $CD3^{+}CD14^{-}CD4^{-}CD8^{+}$ conventional T cells were manually gated in FlowJo. **(a)** FlowSOM clusters (identified in Figure 4) were obtained based on CD45RA, CCR7, 4-1BB, CD25, CTLA-4, HLA-DR, IFNγ, IL-2, IL-4, IL-6, IL-10, IL-17a, PD-1, RORγt, T-BET, TNFα. Expression of each marker on each FlowSOM cluster is shown in histogram format and **(b)** imposed on a tSNE representation.

Supplementary figure 4

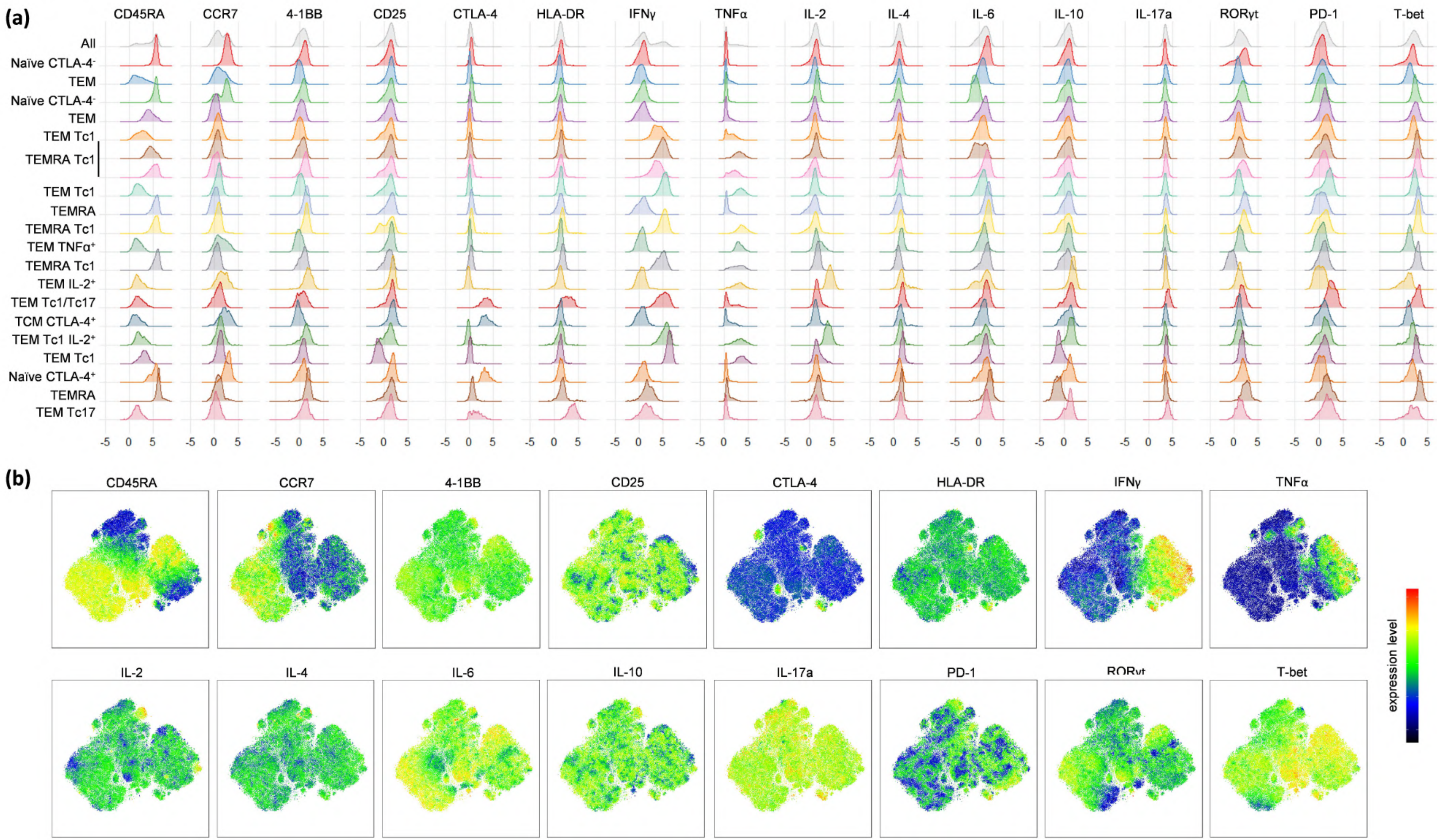

Supplement: Supplementary file 1 [file CTI2-9-e1204-s001.pdf]
